# The physical properties and photocatalytic activities of green synthesized ZnO nanostructures using different ginger extract concentrations

**DOI:** 10.1038/s41598-024-52455-z

**Published:** 2024-01-23

**Authors:** Maryam Aliannezhadi, Seyedeh Zahra Mirsanaee, Mohaddeseh Jamali, Fatemeh Shariatmadar Tehrani

**Affiliations:** Faculty of Physics, Semnan University, PO Box: 35195-363, Semnan, Iran

**Keywords:** Applied physics, Environmental sciences

## Abstract

The green synthesis method which is aligned with the sustainable development goals (SDGs) theory, is proposed to synthesize ZnO nanoparticles using ginger extract to treat the acidic wastewater and acidic factory effluent as a current challenge and the effects of the concentration of extracts on the synthesized ZnO nanostructures are investigated. The results declare that the single-phase hexagonal ZnO is formed using ginger extract concentration of less than 25 mL and the crystallite size of green synthesized ZnO NPs increased with increasing the concentration of ginger extract. Also, the significant effects of ginger extract concentration on the morphology of nanoparticles (nanocone, nanoflakes, and flower-like) and the particle size are demonstrated. The low concentration of ginger extract leads to the formation of the ZnO nanoflakes, while the flower-like structure is gradually completed by increasing the concentration of the ginger extract. Furthermore, significant changes in the specific surface area (SSA) of the samples are observed (in the range of 6.1–27.7 m^2^/g) by the variation of ginger extract concentration and the best SSA is related to using 10 mL ginger extract. Also, the strong effect of using ginger extract on the reflectance spectra of the green synthesized ZnO NPs, especially in the UV region is proved. The indirect (direct) band gap energies of the ZnO samples are obtained in the range of 3.09–3.20 eV (3.32–3.38 eV). Furthermore, the photocatalytic activities of the samples for the degradation of methylene blue indicate the impressive effect of ginger extract concentration on the degradation efficiency of ZnO nanoparticles and it reaches up to 44% and 83% for ZnO NPs prepared using 5 mL ginger extract in a pH of 4.3 and 5.6, respectively. This study provided new insights into the fabrication and practical application of high-performance ZnO photocatalysts synthesized using ginger extract in degrading organic pollutants in an acidic solution.

## Introduction

The synthesis of nanomaterials such as metal nanoparticles, metal oxide semiconductors, and nanocomposites using green synthesis methods has emerged as an environmentally friendly approach^1,2^. These methods have employed natural resources, including plant extracts and microorganisms as reducing or stabilizing agents during the synthesis process^3–5^ and provide various advantages over conventional chemical synthesis methods, such as affordability, non-toxicity, biocompatible, sustainable, friendly, cost-effective, and the capability to produce nanostructures with desirable size, shape, and morphology^5–7^. The green synthesis method aligns with the sustainable development goals (SDGs) theory through responsible consumption, biocompatibility, less toxicity, contributes to clean and less energy usage, and supports goals related to environmental conservation^5^.

Among the metal oxide semiconductors, zinc oxide (ZnO) nanostructures have received significant attention due to their unique properties and application diversity, particularly in photocatalysis activity^8–11^. ZnO has emerged as a groundbreaking photocatalyst, captivating the attention of researchers for its exceptional properties and versatile applications in optoelectronics. Its unique characteristics make it an attractive material, spurring ongoing research endeavors aimed at further understanding, enhancing, and optimizing its potential for diverse optoelectronic applications^12,13^. The green synthesis of ZnO nanostructures typically requires the reduction of zinc precursors in the presence of a green reducing agent which can be derived from leaves, stems, or fruits because of owning rich content of bioactive compounds, such as polyphenols, flavonoids, and alkaloids^14,15^. Different plant extracts like *Opuntia humifusa* fruit extract, *Actinidia deliciosa* fruit peel extract, *Mentha longifolia* leaf extract, and *Eucalyptus globulus* extract have been employed to synthesize ZnO nanoparticles by the green method^16–19^.

The green synthesized ZnO nanostructures have been exploited for the degradation of different dyes^20–24^. For example, *Averrhoe carrambola fruit* extract and zinc nitrate have been used to synthesize ZnO nanoparticles for the degradation of *Congo red* dye under UV–visible light^25^. In other research, ZnO nanoparticles have been obtained via the microwave-assisted green synthesis method using different volumes of *bael *(*Aegle marmelos*)* juice* and zinc nitrate hex-hydrate as a precursor and these ZnO nanoparticles with a surface area of about 26 m^2^/g have been obtained as an eligible candidate for photocatalytic, antimicrobial, and antioxidant utilizations^26^. Also, dye degradation and antibacterial performance of green synthesized ZnO nanoparticles [synthesized using *date pulp waste* (DPW)] by *Phoenix dactylifera* waste as a bio-reductant have been studied, and a rapid decomposition rate with high degradation efficiencies of methylene blue (MB) and eosin yellow dyes were obtained using the irradiation of their synthesized ZnO under UV–visible light^27^. In another study, the ZnO nanoparticles have been synthesized using different concentrations of *Camellia sinensis* extract and calcined at 400 °C for 2 h to use as a photocatalyst agent for MB degradation under UV–visible light, and an MB photodegradation of 84% was provided by the NPs at the irradiation time of 120 min^28^.

Environmental and human health risks derived from toxic heavy metal ions and nitrate have promoted the investigation of different potential solutions, including photocatalysis. Photocatalysis is a promising technique for water purification and it offers the potential to utilize light as a sustainable energy source and operates by utilizing a semiconductor that is excited by light with an energy higher than its band gap which leads to generating electron–hole pairs and then participating in redox reactions^29^. Therefore, the photocatalytic activity of ZnO nanostructures synthesized by green methods is affected by several factors, including the size, shape, and morphology of the nanostructures. Also, green synthesis of ZnO nanostructures can allow the production of ZnO NPs with specific properties and improvements like enhanced photoluminescence emission, enhanced light absorption, providing NPs with high specific surface area, enhanced photocatalytic performance, etc.^30–32^. So, in the current study, the green synthesis method using ginger extract as a reducing agent is proposed and employed to fabricate the ZnO nanostructures in one step and without additives or post-heat treatment. Ginger possesses both antioxidant and anti-inflammatory properties. The phenolic compounds in ginger are mainly *gingerols*, *shogaols*, and *paradols*. In fresh ginger, *gingerols* are the major polyphenols that make it a suitable candidate for reducing agents^33,34^. Then, the effects of different concentrations of the ginger extract on the properties of the green synthesized ZnO are investigated and the governing mechanisms for growing the NPs are explained. After that, synthesized nanocone, nanoflakes, imperfect flower-like, and hierarchical flower-like ZnO samples are established for water treatment from MB dye under UV-light irradiation to evaluate the photocatalytic activities of the NPs and their abilities to use in water treatment. The results declare a significant effect of ginger extract on the features of the provided ZnO NPs and improvement in several characteristic parameters of the NPs compared to the chemically synthesized ZnO nanoparticles.

## Materials and methods

To synthesize the ZnO nanostructures (NSs) using the green synthesis method by ginger extract, chemical components were purchased from Merk company and fresh ginger was provided from the south tropical regions of Iran purchased from the market and extracted to synthesize ZnO nanoparticles using the green method. The fresh ginger was washed several times with deionized water and then dried at room temperature. The use of plants in the present study complies with international, national, and/or institutional guidelines. After that, 2.5 g of grounded ginger was brewed in 50 mL of deionized water on the magnetic stirrer at 70 °C for 30 min. After brewing, the mixture was sonicated at the power of 100 W for 30 min and then the ginger extract was filtered and separated by centrifugation. To study the effect of the extract concentration on the properties of the green synthesized ZnO, the different volumes of the prepared extract including 5 mL, 10 mL, 15 mL, and 25 mL were selected to synthesize ZnO nanoparticles.

4.39 g zinc acetate (ZnC_4_H_6_O_4_) was dissolved with 50 mL ethanol under vigorous stirring and then, 50 mL of the ginger extract with a given concentration was added to the initial solution. The pH value of the solution was adjusted at 11 using sodium hydroxide (NaOH, 2 M). After continuous stirring for 2 h, the solution was sonicated at 70 °C with the power of 100 W for 30 min and the solution was put in the oven at 70 °C for 18 h. Finally, the obtained product was washed with deionized water and ethanol several times and dried at 70 °C for 12 h and then the synthesized samples were labeled according to the volume of ginger extract as ZnG0 (without ginger extract), ZnG5, ZnG10, ZnG15, and ZnG25 and characterized to find information about the synthesized powder.

The structural properties of samples like chemical composition, chemical bonds, and crystallinity were investigated using X-ray diffraction (XRD) (D8-Advance diffractometer employing CuKα radiation, fabricated by Brucker), energy dispersive X-ray spectroscopy (EDX) (MIRA3, fabricated by Tescan) and Fourier transforms infrared (FTIR) (Takram Pluse N1-541, Teksan Co), respectively. Field emission scanning electron microscopy (FESEM) was used to specify the morphology of products (MIRA3, fabricated by Tescan). The specific surface areas of ZnO nanostructures were measured using the N_2_ adsorption–desorption isotherms analysis and Brunauer–Emmett–Teller (BET) method (Belsorb mini system). Also, the optical features of the green synthesized sample were studied using diffuse reflectance spectroscopy (DRS) (Avaspec-2048-TEC device), and UV–Vis transmittance spectroscopy (Shimadzu 1800 UV-visible165-PC double-beam spectrophotometer). Finally, the photocatalysis activities of samples were estimated in the acidic methylene blue solution under UV light at 380 nm by measuring the degradation of methylene blue (MB).

## Results and discussion

### Chemical elements

**Figure 1:**
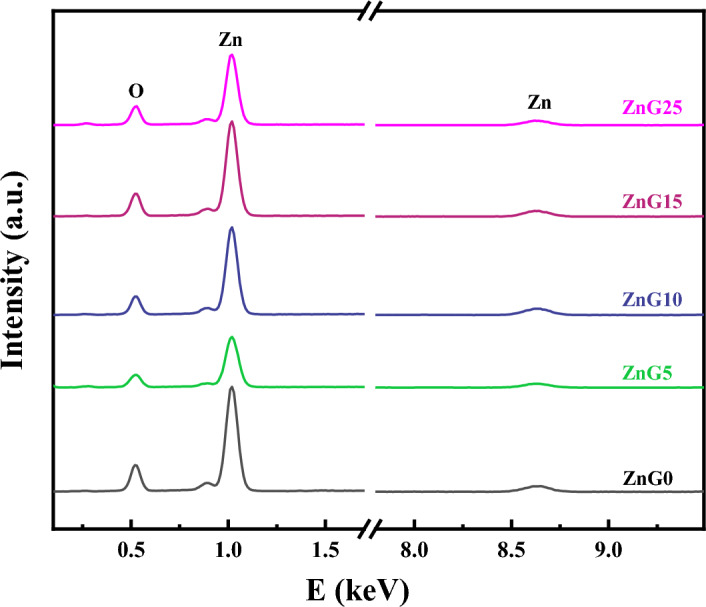
EDX spectra of the ZnO nanostructures synthesized using different concentrations of ginger extract.

EDX analysis was performed on the green synthesized samples to determine the exact elemental composition of them and their EDX spectra are shown in Fig. 1 which indicates that the synthesized samples contain Zn and O elements and confirms the purity of the green synthesized samples. The atomic and weight percentages of existing elements are listed in Table 1. The atomic percentage ratio of Zn to O is approximately 1:1 ratio for all green synthesized samples, indicating that the green synthesized ZnO samples have nearly stoichiometric compositions.

**Table 1:** The EDX results of the ZnO nanostructures synthesized using different concentrations of ginger extract.

Samples	Zn		O	
	A%	W%	A%	W%
ZnG0	41.20	74.11	58.80	25.89
ZnG5	44.90	76.90	55.10	23.10
ZnG10	44.64	76.72	55.36	24.28
ZnG15	41.89	74.66	58.11	25.34
ZnG25	40.42	73.49	59.58	26.51

### Crystallinity

**Figure 2:**
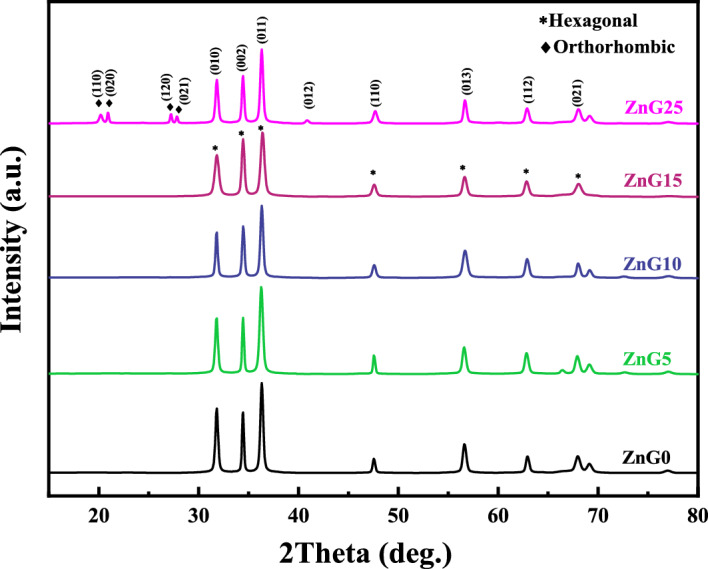
XRD patterns of the ZnO nanostructures synthesized using different concentrations of ginger extract.

The XRD analysis was applied to the green synthesized samples to investigate the crystalline phase and size of the formed structures and the XRD patterns of samples are depicted in Fig. 2, which indicate the good crystalline nature of the samples. Three main diffraction peaks can be observed in all the samples located at *2θ* = 31.77°, 34.37°, and 36.39° which are attributed to (010), (002), and (011) facets of the hexagonal phase ZnO (h-ZnO), respectively. The XRD results declare that all green synthesized samples except ZnG25 have only XRD peaks related to h-ZnO (wurtzite structure) and they formed in a single phase. However, the ZnG25 sample exhibits extra XRD peaks located at *2θ* = 20.18°, 20.90°, 27.70° and 27.80° which are assigned to (110), (020), (120), and (021) planes of the orthorhombic phase Zn(OH)_2_ structure and means the sample was formed in two phases. The phase percentage (%) of the ZnG25 sample is 70:30 for hexagonal: orthorhombic phases obtained using Maud software. Appearing orthorhombic phase by increasing the concentration of the ginger extract can be explained as follows: the ginger extract contains phenolic compounds, volatile oils, polysaccharides, etc. which act as reducing agents, and the functional groups in the phytochemical compounds can chelate Zn ions and provide nucleation sites for creating specific crystal structures. So, the change in the concentration of the different components (bioactive molecules) in plant extract may affect the crystal phase of the synthesized nanoparticles, and crystal symmetry breaking of wurtzite happens in the ZnG25 sample synthesized due to high ginger concentration or high biomolecules concentration used in the synthesis process. The standard cards, lattice parameters, density, and crystallite size of green synthesized ZnO samples are listed in Table 2. Similar phases have different standard cards due to differences in lattice parameters and densities.

**Table 2:** The crystallite properties of the ZnO nanostructures synthesized using different concentrations of ginger extract.

Samples	Standard cards	Lattice parameters (Å)	Density (g/cm3)	Crystallite size (nm)
ZnG0	01-089-0511	a = b = 3.2490, c = 5.2052	5.68	86
ZnG5	01-076-0704	a = b = 3.2530, c = 5.2130	5.66	42.77
ZnG10	01-089-0511	a = b = 3.2490, c = 5.2052	5.68	66.96
ZnG15	01-089-0510	a = b = 3.2488, c = 5.2054	5.68	93.33
ZnG25	01-089-0510 (70%)	a = b = 3.2488, c = 5.2054	5.68	120.31
	01-089-0138 (30%)	a = 4.9050, b = 5.1430 c = 8.4730	3.09	183.33

The crystallite size of synthesized h-ZnO samples is calculated using the Williamson–Hall equation^35^ and it is estimated ~ 86 nm, 43 nm, 67 nm, 93 nm, and 120 nm for ZnG0, ZnG5, ZnG10, ZnG15, and ZnG25 samples, respectively, which means the low concentration of ginger extract leads to smaller crystallite size. Also, the crystallite size of the orthorhombic phase of ZnG25 is ~ 183.33 nm.

### Chemical bonds

**Figure 3:**
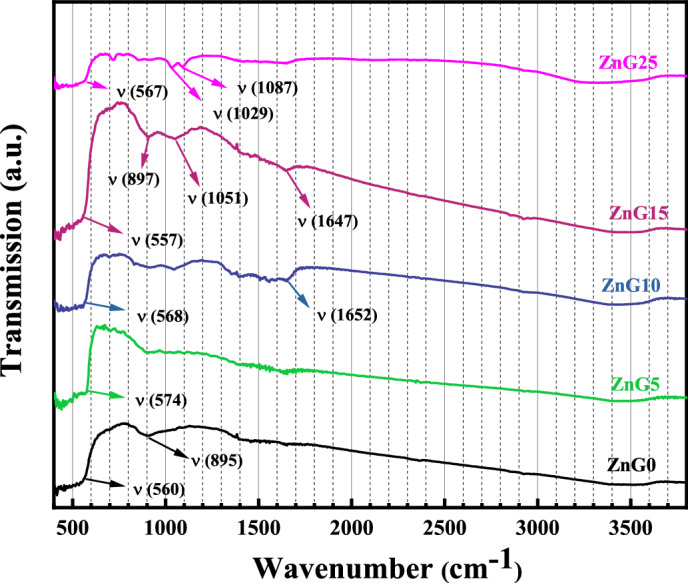
FTIR spectra of the ZnO nanostructures synthesized using different concentrations of ginger extract.

The FTIR spectroscopy was performed to determine the chemical bonding structure of the green synthesized ZnO samples and the FTIR spectra are displayed in Fig. 3. All samples have similar FTIR absorption peaks which prove the existence of the same chemical bonds in the green synthesized ZnO samples produced by different concentrations of ginger extract. The bands located in the range of 400–750 cm^−1^ belong to the Zn–O stretching mode^36^. Generally, the band between 800 and 1600 cm^−1^ is attributed to the organic compounds^37^, and the band at 900–1200 cm^−1^ is associated with the stretching mode of the internally bonded C–H band^38^. The observed bands at around 1650 cm^−1^ and 1050 cm^−1^ are linked with the C=O stretching mode and phenolic group, respectively^39^. Furthermore, C–H stretching mode and C–O stretching mode are detected at about 3000 and 1087 cm^−1^, respectively^25^. Also, The organic compounds participate during the green synthesis process are likely increased with the increment of the extract volume or other word extract concentration in the synthesis process and increasing the phenolic and organic bonds during the formation of ZnO nanoparticles can lead to change in vibration modes which can observed in FTIR spectra of the samples. For example, the peak located at 895 cm^−1^ which is related to C–H vibration mode^40^, splits into two peaks with increasing the extract volum and shift toward higher wavenumbers due to crystallite size enhancement^41^.

### Morphology

**Figure 4:**
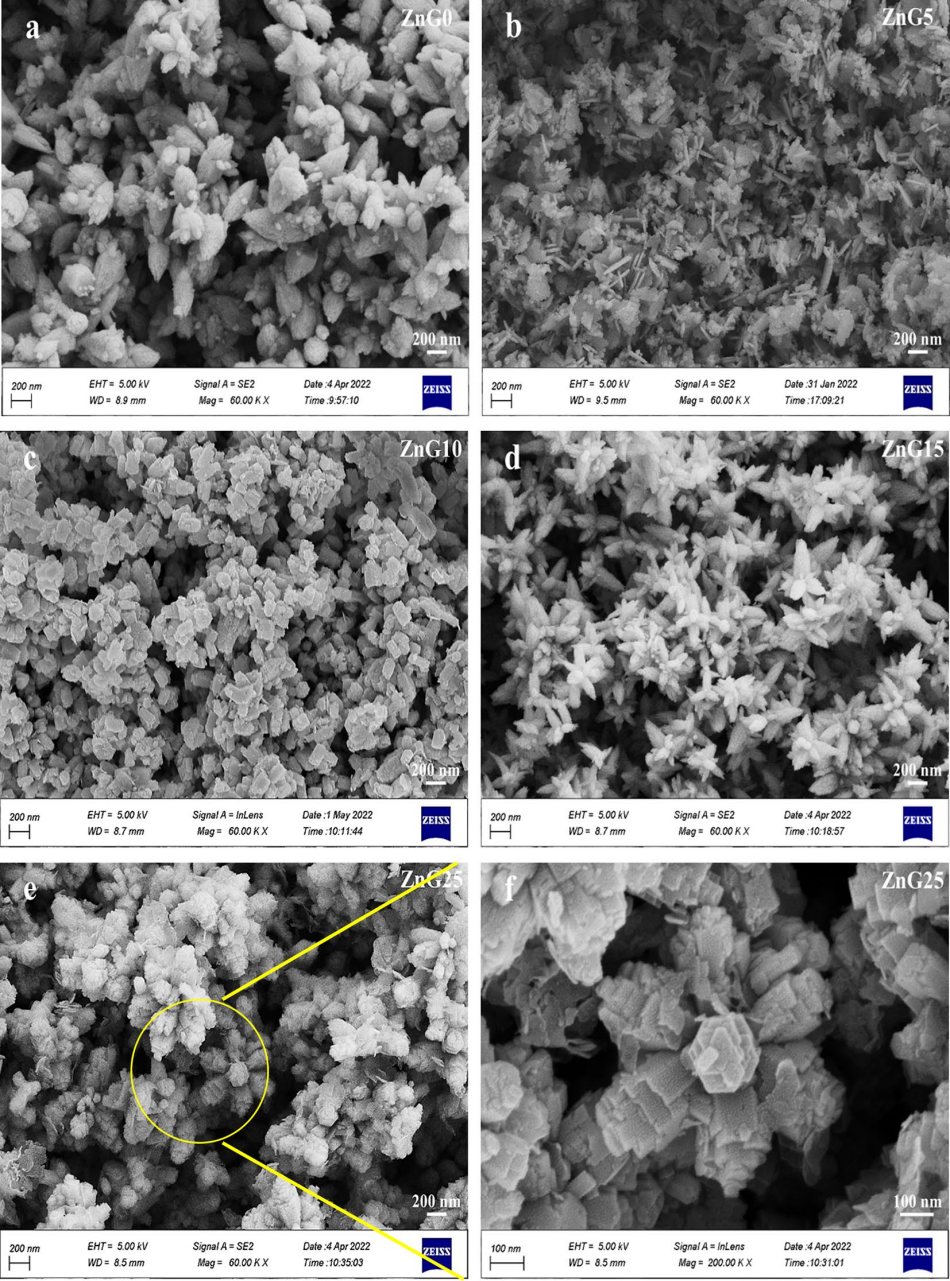
FESEM images of the ZnO nanostructures synthesized using different concentrations of ginger extract: (**a**) ZnG0, (**b**) ZnG5, (**c**) ZnG10, (**d**) ZnG15, and (**e**,**f**) ZnG25 samples with different magnifications.

The morphological characteristics of the green synthesized ZnO samples with different concentrations of ginger extract were studied by FESEM and the results are depicted in Fig. 4. Using Digimizer software and fitting a log–normal function as mentioned in references^42–44^ declare that uniform particles with an average diameter of 200 ± 4.4 nm were created in the absence of ginger extract (Fig. 4a). As seen in Fig. 4b, the addition of ginger extract at the initial concentration (ZnG5) leads to the formation of relatively uniform nanoflakes. The calculated average thickness of nanoflakes is ~ 54 ± 0.6 nm. Also, agglomerated particles with an average diameter of 103 ± 0.9 nm are observed in Fig. 4c for the ZnG10 sample. The flower-like particles can be seen for the ZnG15 sample with an average diameter of about 93 ± 2 nm (Fig. 4d) and the hierarchical flower-like structures appear in the FESEM image of ZnG25 (Fig. 4e,f) which means the self-assembled flower-like ZnO structures are completed by increasing the volume of ginger extract up to 25 mL. To better understand the morphology of the ZnG25 sample, a higher magnification FESEM image of the ZnG25 sample is shown in Fig. 4f which displays the hierarchical flower-like structure composed of adhered plates with an average petals size of 148 ± 1.5 nm. The results demonstrate that the concentration of ginger extract is a very effective quantity on the morphology of green synthesized ZnO nanostructures.

In general, different morphologies and different sizes of ZnO nanoparticles can be obtained using the green method, and some of them are mentioned to compare with our results. Hexagonal particles were formed during the green synthesis of ZnO using *Mehendi* extract with a particle size of 75 nm^45^. Also, Robles et al. synthesized the ZnO nanoparticles with more agglomeration by the increase in *Hibiscus sabdariffa* extract concentration^46^. Furthermore, the synthesis of self-assembled ZnO nanoparticles with an average particle size of 15 nm has been reported by the green method and *Cocos nucifera* leaf extract^47^. It means the morphology of the synthesized ZnO nanoparticles is affected significantly by the synthesis conditions including the type and amount of plant extracts used in the synthesis.

### Growth mechanism

The growth mechanism of synthesized ZnO nanostructures can be explained according to the following chemical reactions^36,48^.

1 $$C_{2} H_{5} OH + (CH_{3} COO)_{2} Zn \to Zn^{2 + } + 2CH_{3} COO + C_{2} H_{5} OH$$

2 $$NaOH \to Na^{ + } + OH^{ - }$$

3 $$Zn^{2 + } + 2OH^{ - } \to Zn(OH)_{2} \leftrightarrow ZnO + H_{2} O$$

Different ions like Zn^2+^, Na^+^, and OH^−^ are formed in the initial solution and the nucleation of ZnO is started after the zinc and hydroxyl ions reaction. Na^+^ ions can play the directing agent role to create various structures^49^ and the generated nuclei will continuously grow over time. On the other hand, in the green synthesis of ZnO nanoparticles using ginger extract, the addition of plant extract helps to more reduction of the zinc ions and it can lead to more stabilization^50^.

In the current green synthesis of ZnO nanostructures, the addition of ginger extract decreases the primary pH value of the solution. For example, the initial pH value of the solution in the synthesis process of ZnG5 was 5.8 which reaches 5.6 after adding ginger extract. The decrease in the solution pH rises by increasing the ginger extract and therefore, more NaOH is required to adjust the pH value at 11 which leads to an increase in the volume of Na^+^ ions and the limitation of particle’s growth along specific directions. Furthermore, increasing the concentration of the ginger extract in the synthesis process means increasing the reducing and capping agents in the precursor solution which leads to enhancing the reduction of metal ions and their subsequent nucleation into metal oxide nanoparticles. Increased nucleation density also can cause nanoparticles to be closer to each other which assists in promoting self-assembly. Also, Ostwald ripening can be promoted by increasing the concentrations of ginger extract due to the increased supersaturation of metal ions in the reaction medium and enhancement of dissolved smaller nanoparticles and increasing their deposit onto larger ones. Hence, the synthesis process may undergo Ostwald ripening and self-assembly phenomena, which can result in an enlargement of particle size and the formation of hierarchical structures^51,52^.

### Specific surface area

**Figure 5:**
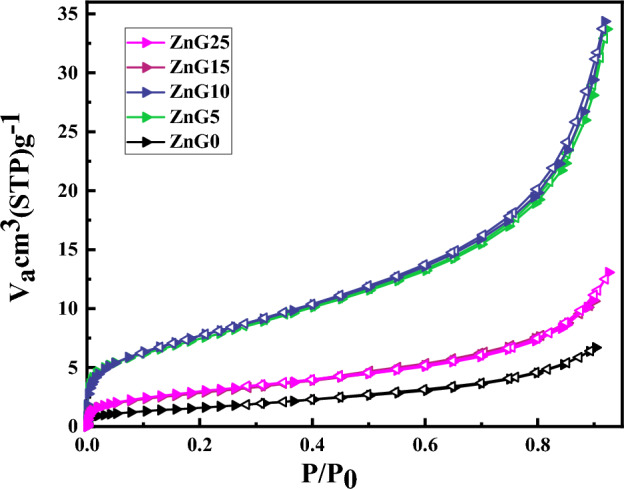
Nitrogen adsorption–desorption isotherm measured for the ZnO nanostructures synthesized using different concentrations of ginger extract.

Nitrogen adsorption–desorption isotherm was measured at 77 K for the green synthesized ZnO samples at different concentrations of ginger extract and the results are presented in Fig. 5. As can be observed in Fig. 5, all green synthesized ZnO nanostructure and ZnG0 can be classified as type IV hysteresis loop (H4 with narrow slit-like pores) behavior according to the IUPAC classification. The specific surface area (SSA) of synthesized ZnO nanostructure is estimated 6.1 m^2^/g, 26.7 m^2^/g, 27.7 m^2^/g, 10.4 m^2^/g, and 10.9 m^2^/g for ZnG0, ZnG5, ZnG10, ZnG15, and ZnG25 samples, respectively, which indicate significant increase in SSA of ZnO nanostructures (> 70%) by using ginger extract in the synthesis process. Partly similar SSA values (≤ 26.8 m^2^/g) have been reported in reference^26^ for green synthesis ZnO nanoparticles using *Indian bael* (*Aegle marmelos*) juice after calcination at 500 °C for 3 h while SSA of the ZnO nanoparticles in the current study obtained in one-step synthesis and without thermal treatment. Also, the SSA of samples can be estimated using crystallite size and density corresponding to the XRD result in Table 2 and equation $SSA = 6/(\rho \times d)$^53^, where *ρ* and *d* are the particle density and average crystallite size of green synthesized ZnO nanoparticles synthesized with different concentrations of ginger extract. The calculated SSA using XRD data are 12.28 m^2^/g, 24.78 m^2^/g, 15.78 m^2^/g, 11.31 m^2^/g, and 8.78 m^2^/g for ZnG0, ZnG5, ZnG10, ZnG15, and ZnG25 samples, respectively. There are no significant differences between estimated specific surface areas obtained by BET and XRD which indicates a close correlation between the crystallite and particle sizes of the synthesized samples and suggests the formation of monocrystalline ZnO nanoparticles.

**Figure 6:**
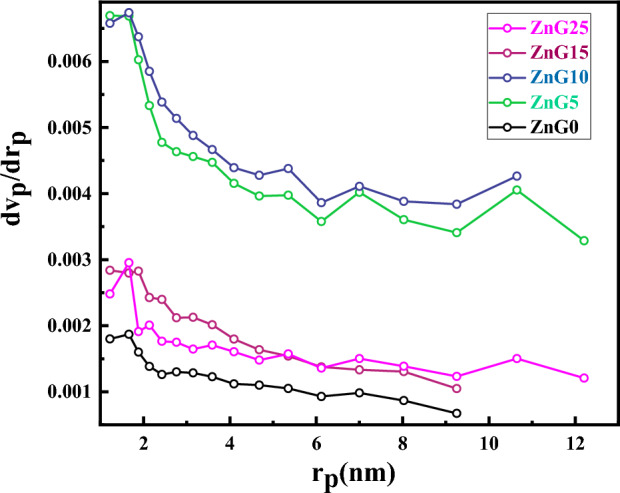
The pore size distribution (BJH curves) of the green synthesized ZnO nanostructures synthesized using different concentrations of ginger extract.

To find some information about the pore size distributions of the green synthesized using ginger extract, Barrett–Joyner–Halenda (BJH) curves of the samples are exhibited in Fig. 6 which indicate all samples have a peak below 2 nm and the average pore size of 1.65 nm, 1.63 nm, 1.67 nm, 1.88 nm, and 1.65 nm for ZnG0, ZnG5, ZnG10, ZnG15, and ZnG25 samples, respectively.

### Optical properties

#### Diffuse reflectance spectroscopy (DRS)

**Figure 7:**
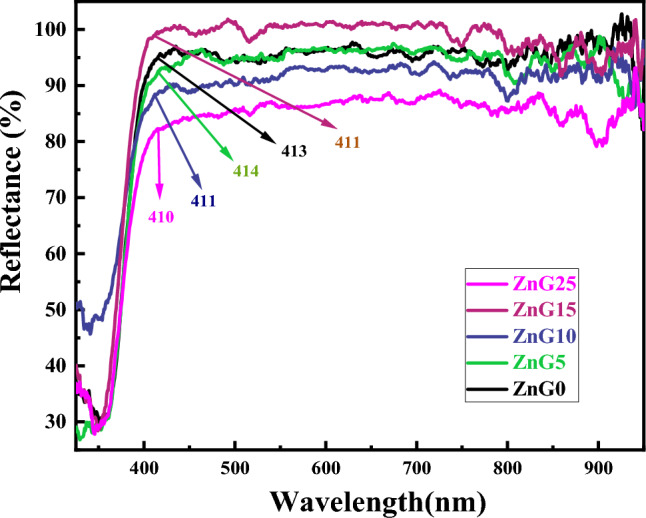
DRS spectra of the ZnO nanostructures synthesized using different concentrations of ginger extract.

To study the optical properties of the samples, diffuse reflectance spectroscopy (DRS) was applied on the green synthesized ZnO nanostructure using different concentrations of ginger extract, and the reflectance spectra of the samples are presented in Fig. 7. It can be seen that using the ginger extract causes a significant change in the reflectance of the ZnO samples especially in the UV region which ZnG0 (blue solid line in Fig. 7) shows a higher reflectance than green synthesized ZnO nanoparticles. Also, the absorbance edge of ZnO samples is slightly shifted with increasing the concentration of ginger extract, and it is located at the wavelength range of 410–413 nm.

**Figure 8:**
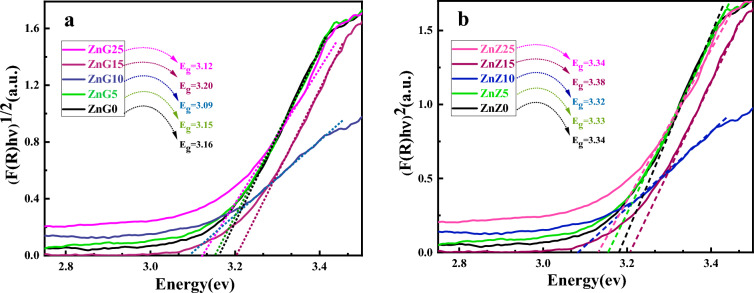
Tauc’s plot of the ZnO nanostructures synthesized using different concentrations of ginger extract to calculate (**a**) indirect and (**b**) direct bandgap energies.

The indirect and direct band gap of ZnO nanostructures are calculated using the Tauc formula ((*F*(*R*)*hν*)^*n*^ = *B*(*E − E*_*g*_))^54^ and Kubelka–Munk function (*F*(*R*) = (*1 – R*^2^)/(*2R*))^55,56^, where *R, B, E,* and *E*_*g*_ are reflectance coefficient, a constant coefficient, photon energy, and band gap energy in Tauc formula, respectively. The indirect (direct) band gap values calculated from the Tauc plots in Fig. 8 are about 3.16 eV (3.34 eV), 3.15 eV (3.33 eV), 3.09 eV (3.32 eV), 3.20 eV (3.38 eV), and 3.12 eV (3.34 eV) for ZnG0, ZnG5, ZnG10, ZnG15 and ZnG25 samples, respectively, which shows slight changes in the indirect (direct) band gap energies of the synthesized ZnO nanostructures with ginger extract concentration. The consistent results for band gap energies of the green synthesized ZnO nanoparticles have been reported in the literature^18,25,57^. In another research, despite the creation of ZnO/ MXene composites, the band gap of pure ZnO was modified from 3.22 to 3.19 eV^58^ which we obtained the band gap without providing composite and with pure ZnO nanoparticles synthesized by the green method.

### Photocatalysis activity

**Figure 9:**
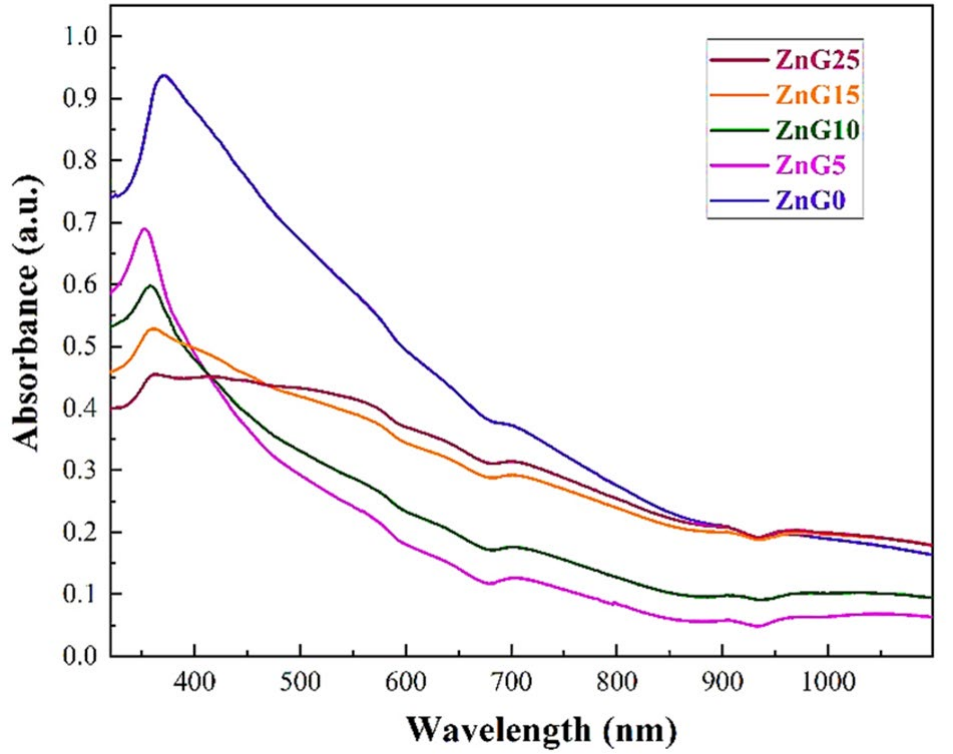
The UV–Vis spectra of the ZnO nanostructures synthesized using different concentrations of ginger extract.

To consider the light absorption of the green synthesized ZnO nanoparticles in a liquid environment, 0.001 g of the nanoparticles were dispersed in 10 mL of DI water, and then the transmittance UV–Vis spectroscopy was applied to the dispersed samples, and the corresponding absorbance spectra of ZnO nanoparticles are illustrated in Fig. 9. The broad peak related to the ZnG0 sample is located at about 360 nm in the UV region which experiences a blue shift after using the ginger extract in the synthesis process. However, a red-shift can be observed in the absorption peak of the ZnO samples with increasing the ginger extract from 5 to 25 mL which can be mainly due to the increase of defects and oxygen vacancies in the lower concentration of ginger extract. Therefore, the UV light with the central wavelength of 380 nm was selected to study the photocatalysis performance of the green synthesized ZnO nanostructures.

**Figure 10:**
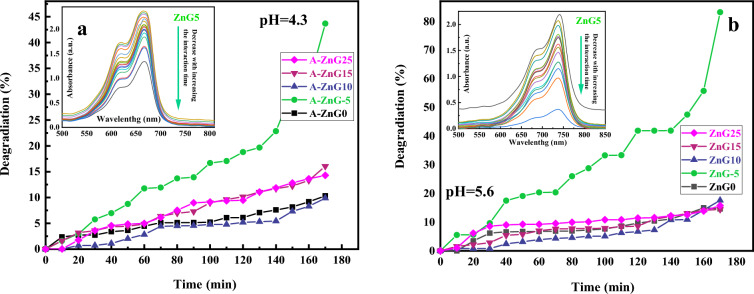
The photodegradation% of MB solutions under irradiation for the ZnO nanostructures synthesized using different concentrations of ginger extract in (**a**), pH 4.3 and (**b**) pH 5.6. The absorbance spectra of MB solution treated by irradiated ZnG5 nanoparticles at different interaction times in pH of 4.3 and 5.6 are plotted in the inset of (**a**,**b**), respectively.

The photocatalytic activities of green synthesized ZnO nanostructures with different concentrations of ginger extract were investigated by studying the degradation of methylene blue (10 ppm) with 10 mg of photocatalyst under UV-light radiation at a duration time of 150 min. Also, since the degradation in acidic conditions has a significant role in the treatment of acidic industrial wastewater as a global environmental challenge and the water purification efficiencies of most nanoparticles are not suitable in the acidic region, and nanoparticles are almost powerless in this region, the pH values of 4.3 and 5.6 is selected to study the photocatalytic activities of synthesized ZnO nanoparticles. The acidic methylene blue solution containing the ZnO NPs was located in a dark condition for 30 min due to the attainment of adsorption–desorption equilibrium between photocatalysis and methylene blue. Then the solution was exposed to continuous UV-light irradiation, and the degradation of the MB was evaluated using the absorption spectra of MB by a UV–Vis spectrophotometer at given times. Then, the efficiency of photocatalytic degradation is calculated using the following formula^40^:

5 $$\eta = \frac{{C_{0} - C}}{{C_{0} }} \times 100$$

where *C*_0_ (mg/L) is the initial concentration of MB, *C* (mg/L) is the final concentration of MB after photodegradation. Treatment of acidic wastewater from factories is a current challenge and most nanoparticles have a low dye removal or dye photodegradation in acidic conditions. As can be observed in Table 3, most studies reported in the dye photodegradation by ZnO nanostructures have been done in neutral and alkaline conditions due to the weakness of the nanoparticles in the acidic pH. So, the photocatalytic activities of the green synthesized ZnO nanoparticles under UV light and in acidic conditions (pH 5.6 and 4.3) were considered to find desirable NPs for this condition and the results are reported in Fig. 10. It can be observed in Fig. 10a,b that the efficiency of all green synthesized ZnO nanoparticles expect ZnG5 in water treatment is low (less than 17% in pH of 5.6 and 4.3) and they are not efficient for the treatment of acidic wastewater of factories. As it can be obtained from the absorbance spectra of MB solution treated by irradiated ZnG5 nanoparticles at different interaction times in pH of 4.3 and 5.6 in the inset of Fig. 10a,b, increasing the interaction time of light and ZnG5 leads to a significant decrease in the MB molecules in the acidic solution and this decreasing trend rise with increasing the solution pH from 4.3 to 5.6. By considering the results of Fig. 10 and Table 3, it can be figured out that the efficiency of ZnG5 in the treatment of acidic water with MB dye under UV irradiation is admirably higher than other reported works in the scientific literature.

**Table 3:** The comparison of MB degradation using the irradiated ZnO nanostructures reported in the scientific literature at different pH values.

Sample	Catalyst mass (mg)	Dye concentration (ppm)	Degradation (%)	pH value	References
Y-ZnO	10	80	90	10	^59^
Ag-ZnO	50	10	92	Neutral	^60^
Ag-ZnO	100	2 × 10^–5^ M	76	Neutral	^61^
Mn-ZnO	30	10	99	10	^62^
ZnO (green synthesis)	25	10	97	Neutral	^63^
ZnO (green synthesis)	200	5 and 15	99	Neutral	^64^
ZnO (green synthesis)	80	10	92	10	^65^
ZnO (green synthesis)	100	32	91	Neutral	^66^
RGO-Ag-ZnO (green synthesis)	50	10	99	8.4	^67^
Ga:ZnO	50	10	95	Neutral	^68^
green synthesized ZnO	10	10	44	4.3	Current study
	10	10	83	5.6

The high efficiency of the ZnG5 sample for the treatment of acidic waters (43% and 83% for pH values of 4.3 and 5.6, respectively) can be understood by its lower crystallite and particle size, higher specific surface area, and high absorbance in the UV region. The high absorbance leads to more absorption of photons and the production of more electron–hole pairs which along with its high SSA leads to the generation of more radicals due to more dye-NPs contact, more active sites, producing more electron–hole pairs, etc.

## Conclusion

To overcome the challenge of treating the acidic wastewater from dyes, the synthesis of ZnO nanostructures using the sol–gel green method as a cost-effective and eco-friendly route was proposed by ginger extract as a rich source of biomolecules for ions reduction, and the effects of ginger concentration on the formed nanoparticles were investigated. The organized study of ZnO nanostructures exhibited the significant effect of ginger extract concentration on the properties of synthesized ZnO NPs. The results have proved the formation of pure ZnO nanostructures and the average crystallite size of the ZnO NPs increased with the enhancement of the extract concentration from 66 to 120 nm. Also, the low concentration of ginger extract led to the formation of the ZnO nanoflakes, while the flower-like structure was gradually completed by increasing the concentration of the ginger extract. The specific surface area (SSA) of 27 m^2^/g with a size distribution peak below 2 nm was obtained for the ZnO nanoparticles synthesized using 5 mL ginger extract (ZnG5). The band gap energies of the green synthesized samples were between 3.09 and 3.20 eV as obtained from DRS data. Also, UV light was selected for the investigation of the photocatalysis activities of the green synthesized ZnO nanoparticles in two different acidic pHs of MB solutions due to the broad absorption peak of the samples in this region. The efficiencies of the green synthesized ZnO NPs for the treatment of acidic water were investigated by considering the photodegradation of MB (10 ppm) under irradiated NPs and the high efficiencies of ZnO nanoparticles synthesized using 5 mL ginger extract (ZnG5) was obtained which were 44% and 83% at a duration time of 150 min in the solution pH of 4.3 and 5.6, respectively. It is an admirable achievement that can solve the current challenge in the treatment of acidic wastewater and acid factory effluent.

## Data Availability

All data included in this paper are available upon request by contact with the contact corresponding author.
